# Low Serum Neutrophil Gelatinase-associated Lipocalin Level as a Marker of Malnutrition in Maintenance Hemodialysis Patients

**DOI:** 10.1371/journal.pone.0132539

**Published:** 2015-07-10

**Authors:** Hirotaka Imamaki, Akira Ishii, Hideki Yokoi, Masato Kasahara, Takashige Kuwabara, Keita P. Mori, Yukiko Kato, Takashi Kuwahara, Masugi Satoh, Kimihiko Nakatani, Yoshihiko Saito, Naohisa Tomosugi, Akira Sugawara, Kazuwa Nakao, Masashi Mukoyama, Motoko Yanagita, Kiyoshi Mori

**Affiliations:** 1 Department of Nephrology, Kyoto University Graduate School of Medicine, Kyoto, Japan; 2 Institute for Advancement of Clinical and Translational Science, Kyoto University Hospital, Kyoto, Japan; 3 Department of Nephrology, Saiseikai Ibaraki Hospital, Osaka, Japan; 4 Misugikai Satou Hospital, Osaka, Japan; 5 First Department of Internal Medicine, Nara Medical University, Nara, Japan; 6 Division of Advanced Medicine, Medical Research Institute, Kanazawa Medical University, Kanazawa, Japan; 7 Department of Nephrology, Osaka Red Cross Hospital, Osaka, Japan; 8 TK Project, Medical Innovation Center, Kyoto University Graduate School of Medicine, Kyoto, Japan; 9 Department of Nephrology, Kumamoto University Graduate School of Medical Sciences, Kumamoto, Japan; 10 TMK Project, Medical Innovation Center, Kyoto University Graduate School of Medicine, Kyoto, Japan; Tokushima University Graduate School, JAPAN

## Abstract

**Background:**

Neutrophil gelatinase-associated lipocalin (NGAL or LCN2) is an iron-transporting factor which possesses various activities such as amelioration of kidney injury and host defense against pathogens. Its circulating concentrations are elevated in acute and chronic kidney diseases and show a positive correlation with poor renal outcome and mortality, but its clinical significance in maintenance hemodialysis (HD) patients remains elusive.

**Methods:**

Serum NGAL levels were determined by enzyme-linked immunosorbent assay in out-patient, Japanese HD subjects. Their correlation to laboratory findings and morbidity (as development of severe infection or serum albumin reduction) was investigated using linear regression analysis and χ^2^ test.

**Results:**

Pre-dialysis serum NGAL levels in HD patients were elevated by 13-fold compared to healthy subjects (n=8, P<0.001). In a cross-sectional study of 139 cases, serum NGAL concentrations were determined independently by % creatinine generation rate (an indicator of muscle mass, standardized coefficient β=0.40, P<0.001), peripheral blood neutrophil count (β=0.38, P<0.001) and anion gap (which likely reflects dietary protein intake, β=0.16, P<0.05). Iron administration to anemic HD patients caused marked elevation of peripheral blood hemoglobin, serum ferritin and iron-regulatory hormone hepcidin-25 levels, but NGAL levels were not affected. In a prospective study of 87 cases, increase in serum albumin levels a year later was positively associated to baseline NGAL levels by univariate analysis (r=0.36, P<0.01). Furthermore, within a year, patients with the lowest NGAL tertile showed significantly increased risk for marked decline in serum albumin levels (≥0.4 g/dl; odds ratio 5.5, 95% confidence interval 1.5–20.3, P<0.05) and tendency of increased occurrence of severe infection requiring admission (odds ratio 3.1, not significant) compared to the middle and highest tertiles.

**Conclusion:**

Low serum NGAL levels appear to be associated with current malnutrition and also its progressive worsening in maintenance HD patients.

## Introduction

Neutrophil gelatinase-associated lipocalin (NGAL, lipocalin 2 or LCN2) was initially purified from neutrophils but with unknown function [1]. X-ray crystallography revealed that NGAL is bound to iron in the presence of organic cofactors which is called siderophores [2]. Thereafter, a number of iron-dependent NGAL activities have been identified [3–5]. As an iron donor, NGAL prevents acute kidney injury (AKI) [6], activates kidney differentiation [7] and modulates cancer metastasis [8, 9]. On the other hand, by iron chelation, NGAL inhibits growth of pathogens including *E*. *Coli* [10], *Mycobacterium tuberculosis* [11] and *Klebsiella pneumoniae* [12]. With respect to regulation of NGAL expression, kidney injury [3, 6, 13], infection [10, 14], inflammation [15], and malignancy [8, 9] are major inducers of expression in epithelial and non-epithelial cells, but the role of iron status itself in regulation of NGAL, especially among end-stage renal disease patients, remains largely unknown.

In AKI, serum and urinary NGAL levels are elevated rapidly, which occurs a few days earlier than increase in serum creatine levels [3, 5, 16, 17]. Among patients in intensive care unit, blood NGAL levels predict requirement of renal replacement therapy and death [18, 19]. Furthermore, in the settings of chronic kidney disease (CKD), blood NGAL levels are correlated to serum creatinine levels [20] and are associated with progression of CKD [21]. However, little is known about clinical significance of blood NGAL levels in hemodialysis (HD) patients. Patients receiving maintenace HD are a unique group of subjects whose clinical parameters are closely monitored in routine practice. Furthermore, their dietary protein intake and muscle mass can be estimated by formulas using blood urea nitrogen (BUN) and creatinine levels before and after HD session [22, 23].

In the present study, we performed comprehensive analysis for cross-sectional association of serum NGAL levels with clinical parameters including indices of iron homeostasis and nutrition among 139 Japanese maintenanse HD patients. Univariate and multivariate linear regression analyses revealed positive correlation between NGAL levels and nutritional markers. Furthermore, as markers of morbidity, we longitudinally investigated development of severe infection within a year and changes in serum albumin levels after a year, since malnutrition increases a risk for occurrence of infection and the serum albumin level, as a representative indicator of nutritional status, is a strong predictor of morbidity and mortality in hemodialysis patients [24, 25]. We also investigated whether serum NGAL levels are altered by iron administration, HD session and passage of blood through the kidneys.

## Results

### Baseline characteristics of patients

Baseline characteristics of 139 out-patient, Asian-Japanese, maintenance HD subjects are shown in Table 1, S1 and S2 Tables. Clinical parameters studied include routine laboratory data and indices of nutrition, iron status and HD efficiency. Pre-dialysis serum NGAL levels in HD patients (mean±SD, 916±345 ng/ml) were elevated approximately by 13-fold compared to healthy subjects (68±15 ng/ml, n = 8, P<0.001). When patients were divided into tertiles of serum NGAL levels, several clinical parameters showed significant difference among groups (Table 1), which were further characterized below. Gender and presence of diabetes mellitus (DM) did not significantly affect serum NGAL levels (male, 899±354, n = 79; female, 937±334, n = 60; cases with DM, 841±291, n = 38; without DM, 944±360 ng/ml, n = 101).

**Table 1:** Baseline characteristics of 139 maintenance HD patients in correlation with tertiles of serum NGAL concentrations.

	Pre-dialysis NGAL tertile			
Baseline characteristics	Low	Medium	High	P for trend
N	46	47	46	
Pre-dialysis NGAL (ng/ml)	583 (500–663)	872 (826–943)	1207 (1130–1423)	<0.001
Post-dialysis NGAL (ng/ml)	417 (331–490)	639 (513–773)	878 (768–1013)	<0.001
NGAL removal ratio (%)	24±16	28±17	29±18	0.37
Age (year)	69±10	64±13	62±11	0.02
Male gender [n (%)]	31 (67%)	20 (42%)	28 (60%)	<0.001
Diabetes [n (%)]	14(30%)	14 (30%)	10 (22%)	0.34
HD period (month)	29 (13–68)	74 (42–140)	102 (53–156)	<0.001
Na (mEq/l)	140±3	139±3	138±3	<0.001
Cl (mEq/l)	105±4	103±3	102±4	<0.001
K (mEq/l)	4.8±0.9	5.0±0.7	5.0±0.8	0.37
Ca (mg/dl)	8.5±0.9	8.7±0.9	8.9±0.8	0.04
Normalized Ca (mg/dl)	8.9±0.8	9.1±0.8	9.1±0.7	0.25
Phosphorus (mg/dl)	4.8±1.4	5.2±1.4	5.8±1.8	0.01
Intact PTH (pg/ml)	162 (97–232)	183 (92–287)	215 (81–336)	0.19
BUN (mg/dl)	61±16	65±13	72±17	0.01
Creatinine (mg/dl)	9.1±2.4	11.0±2.4	12.2±2.6	<0.001
UA (mg/dl)	6.4±1.4	6.7±1.2	7.2±1.3	0.03
Total protein (g/dl)	6.2±0.4	6.4±0.5	6.6±0.4	<0.001
Albumin (g/dl)	3.7±0.4	3.7±0.4	3.9±0.3	<0.01
Choline esterase (IU/l)	197±55	219±58	227 ±71	0.06
Total cholesterol (mg/dl)	146±33	156±30	164±50	0.06
Triglyceride (mg/dl)	76 (63–101)	96 (63–172)	106 (78–146)	0.05
CRP (mg/dl)	0.10 (0.05–0.37)	0.10 (0.05–0.26)	0.12 (0.07–0.30)	0.21
Base excess (mEq/l)	-3.9±3.3	-4.4±3.0	-6.4±3.7	<0.01
Anion gap (mEq/l)	19.7±2.3	20.4±2.7	22.4±3.0	<0.001
White blood cell (/μl)	4912±1456	5346±1639	7146±2054	<0.001
Neutrophil (/μl)	3187±1108	3691±1246	5109±1892	<0.001
Red blood cell (x10^6^/μl)	3.34±0.56	3.42±0.50	3.51±0.47	0.28
Hemoglobin (g/dl)	10.2±1.4	10.0±1.4	10.7±1.4	0.03
Hematocrit (%)	32.0 (29.5–35.2)	31.9 (28.3–34.7)	33.9 (30.8–35.9)	0.31
Platelet (x10^3^/μl)	152±47	175±63	197±59	<0.01
Ferritin (ng/ml)	126 (43–225)	80 (33–184)	148 (52–120)	0.18
Iron (mg/dl)	68±31	65±28	63±38	0.70
TIBC (mg/dl)	235±33	258±49	248±57	0.08
UIBC (mg/dl)	168±45	191±64	184±63	0.15
TSAT (%)	29.0±14.6	26.2±13.1	26.4±14.6	0.53
%CGR (%)	81±27	106±23	110±24	<0.001
nPCR (g/kg/day)	0.83±0.20	0.91±0.15	0.96±0.20	<0.001
Kt/Vsp	1.31±0.32	1.49±0.29	1.41±0.29	0.02

Biochemical analysis was carried out using pre-dialysis serum, unless specified. See S1 Table for other clinical parameters. Values are expressed as mean±SD, median (interquartile range) or number (%). Trend for differences among serum NGAL tertiles was compared by ANOVA for continuous variables and by Kruskal-Wallis test for dichotomous and non-normal variables, respectively. PTH, parathyroid hormone; BUN, blood urea nitrogen; UA, uric acid; CRP, C-reactive protein; TIBC, total iron binding capacity; UIBC, unsaturated iron binding capacity; TSAT, transferrin saturation; CGR, creatinine generation rate; nPCR, normalized protein catabolic rate; Kt/Vsp, single-pool Kt/V.

### Univariate analysis of serum NGAL levels

In a cross-sectional study, we examined association of baseline serum NGAL levels with above mentioned parameters, using univariate linear regression analysis (S3 Table). Among the parameters examined, peripheral blood neutrophil count, white blood cell (WBC) count, anion gap, serum creatinine and % creatinine generation rate (%CGR) exhibited the strongest correlation coefficients (r) with serum NGAL levels, which were >0.48, respectively (P<0.001, Table 2). None of indices for iron homeostasis, including red blood cell count, blood hemoglobin, hematocrit, reticulocyte count, serum ferritin, iron, total iron binding capacity, unsaturated iron binding capacity and transferrin saturation were significantly associated with NGAL levels (P≥0.05, respectively, S3 Table), suggesting that systemic iron abundance does not affect serum NGAL levels.

**Table 2:** Clinical parameters showing significant correlations with baseline pre-dialysis serum NGAL levels by univariate linear regression analysis.

	r	P
Neutrophil	0.53	< 0.001
White blood cell	0.52	< 0.001
Anion gap	0.51	< 0.001
Creatinine	0.51	< 0.001
%CGR	0.49	< 0.001
HD period	0.47	< 0.001
pH	-0.37	< 0.001
Platelet	0.35	< 0.001
Na	-0.33	< 0.001
nPCR	0.33	< 0.001
BUN	0.33	< 0.001
Cl	-0.32	< 0.001
Base excess	-0.32	< 0.001
Albumin	0.29	< 0.001
Age	-0.29	< 0.001
Phosphorus	0.29	0.001
Total protein	0.29	0.001
HCO_3_^-^	-0.28	0.001
Ca	0.27	0.002
UA	0.26	0.003
Choline esterase	0.23	0.01
Normalized Ca	0.22	0.01
Total cholesterol	0.20	0.02
Kt/Vsp	0.20	0.02

Among clinical parameters shown in Table 1, parameters which had significant correlations to serum NGAL levels are shown (P<0.05, respectively). N = 139. Correlations of the rest of parameters are shown in S3 Table.

### Multivariate analysis of serum NGAL levels

**Fig 1:**
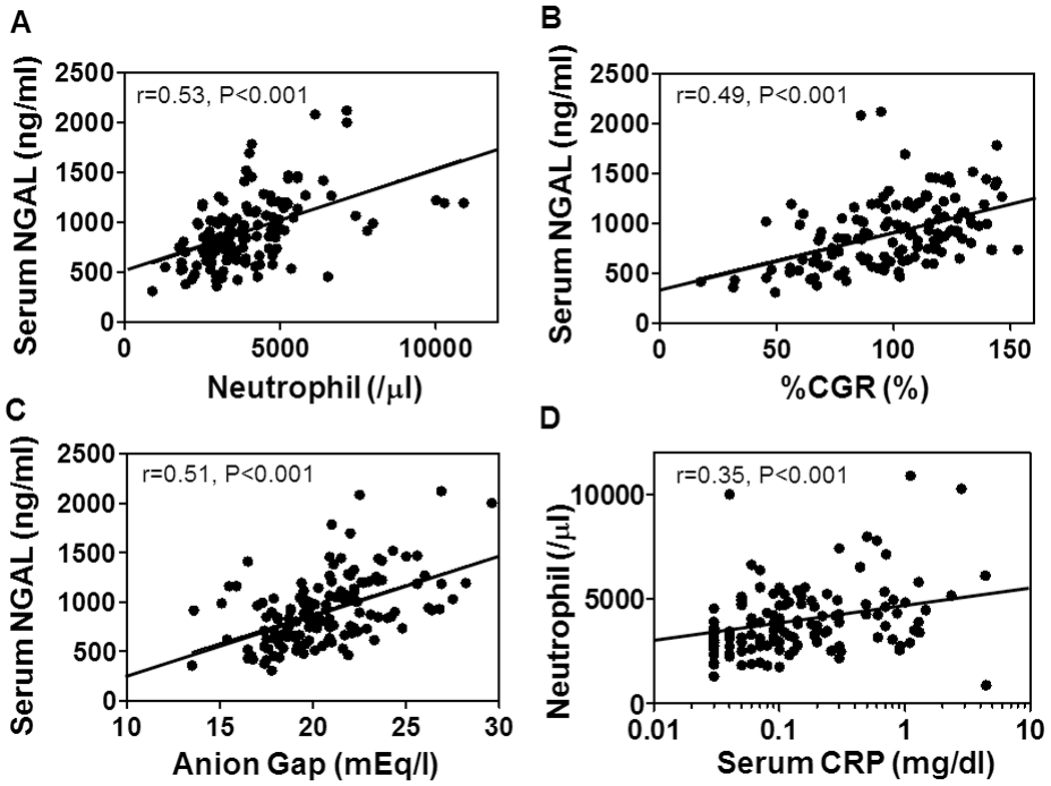
Linear correlation of baseline pre-dialysis serum NGAL levels with clinical parameters. Correlations of serum NGAL level with (**A**) peripheral blood neutrophil count, (**B**) % creatinine generation rate (%CGR) and (**C**) anion gap are shown. (**D**) Correlation between neutrophil count and serum C-reactive protein (CRP) level is also shown. Linear line in each graph shows regression line. N = 139.

To carry out multivariate linear regression analysis for baseline serum NGAL levels, we entered not only age and gender but also all the above 5 variables tightly correlated to serum NGAL levels, since it was difficult to pick up representative variables among them. Through this analysis, %CGR, neutrophil count and anion gap were selected as independent variables determining serum NGAL levels (Table 3, Fig 1A–1C).

**Table 3:** Determinants of baseline serum NGAL levels by multivariate linear regression analysis.

Variable	Standardized coefficient (β)	P
%CGR	0.40	<0.001
Neutrophil	0.38	<0.001
Anion gap	0.16	<0.05

The above 3 variables explained 42% of the expected NGAL value (adjusted r^2^). Variables entered but excluded by stepwise analysis were age, gender, serum creatinine, and blood WBC count. N = 139.

Next, we investigated clinical implication of the above findings (Fig 1 and S4 Table). Blood neutrophil count showed strong correlation with WBC count (r = 0.92, P<0.001) and serum CRP level (r = 0.35, P<0.001), suggesting that neutrophil count is a marker of bone marrow myeloid activity and inflammatory status (S4 Table, Fig 1D). %CGR, which is known as an indicator of muscle mass [23], exhibited the strongest correlation to serum creatinine (r = 0.84, P<0.001). Anion gap and normalized protein catabolic rate (nPCR) have been reported to be highly correlated to dietary protein intake in HD patients [22, 26], and they were associated well to each other (r = 0.50, P<0.001)[26]. Furthermore, serum NGAL levels were significantly correlated to other markers of nutrition such as serum albumin (r = 0.29, P<0.001), choline esterase (r = 0.23, P = 0.01) and phosphorus levels (r = 0.29, P = 0.001) (Table 2). These findings suggest that muscle mass, protein intake, myeloid activity and inflammatory status are the major determinants of serum NGAL levels in steady-state maintenance HD patients.

### Impact of HD efficiency, HD period and age upon serum NGAL

**Fig 2:**
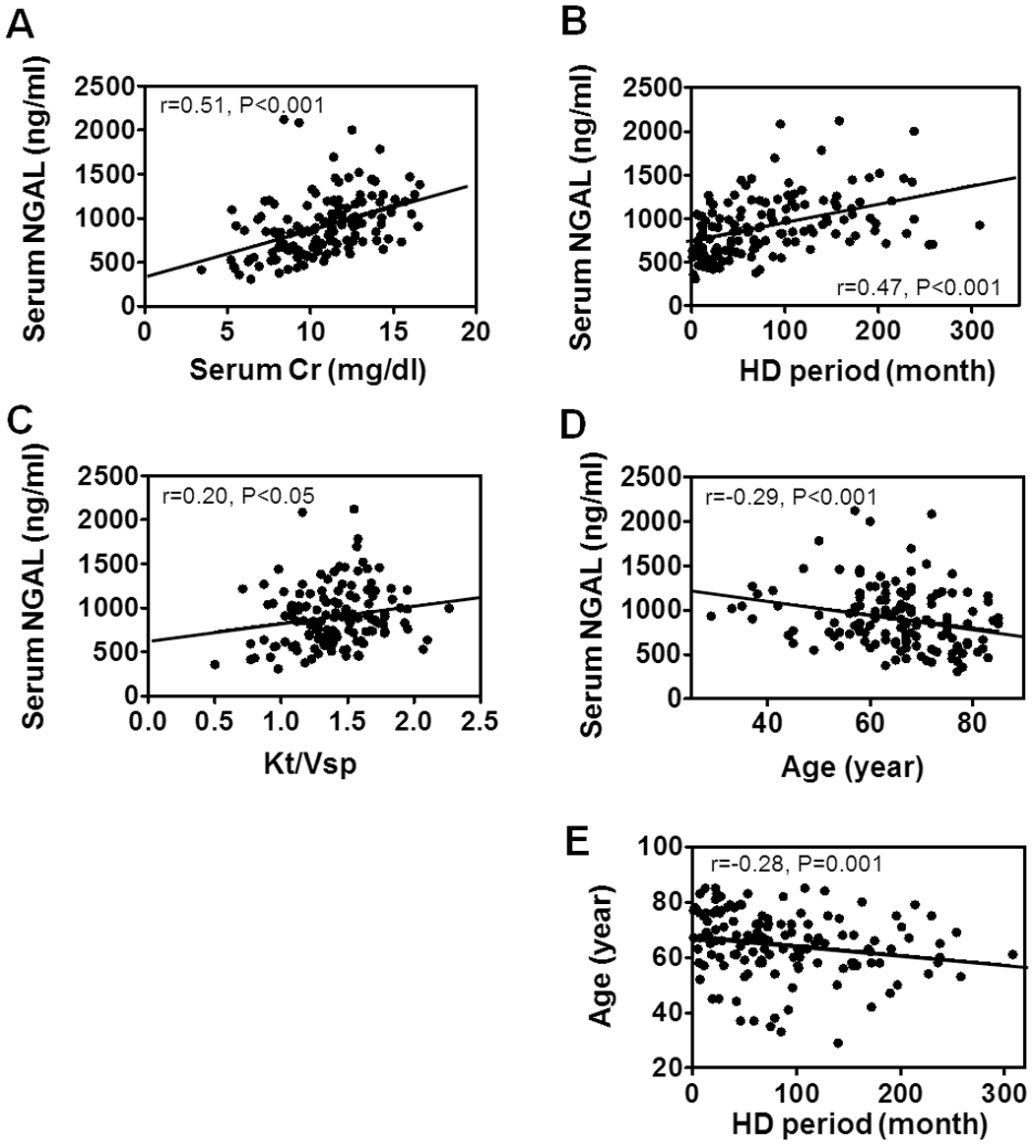
Linear correlation of baseline pre-dialysis serum NGAL levels with serum creatinine, dialysis efficiency, age and HD period. Correlations with (**A**) creatinine (Cr), (**B**) HD period, (**C**) single-pool Kt/V (Kt/Vsp), and (**D**) age are shown. (**E**) Correlation between HD period and age. N = 139.

Since serum NGAL levels were positively correlated to serum creatinine levels (r = 0.51, P<0.001) and to HD period (r = 0.47, P<0.001), we examined whether elevated NGAL was caused by low dialysis efficiency (Table 2, Fig 2A and 2B). Oppositely, HD efficiency calculated as single-pool Kt/V (Kt/Vsp) was positively associated with NGAL levels (r = 0.20, P<0.05, Fig 2C). Furthermore, serum NGAL levels were negatively correlated to age (r = -0.29, P<0.001, Fig 2D) and age was negatively correlated to HD period (r = -0.28, P = 0.001, Fig 2E). These findings suggest that patients with long HD history had elevated serum NGAL levels because they contained relatively younger subjects whose nutritional conditions were good.

### Impact of iron administration upon serum NGAL

**Fig 3:**
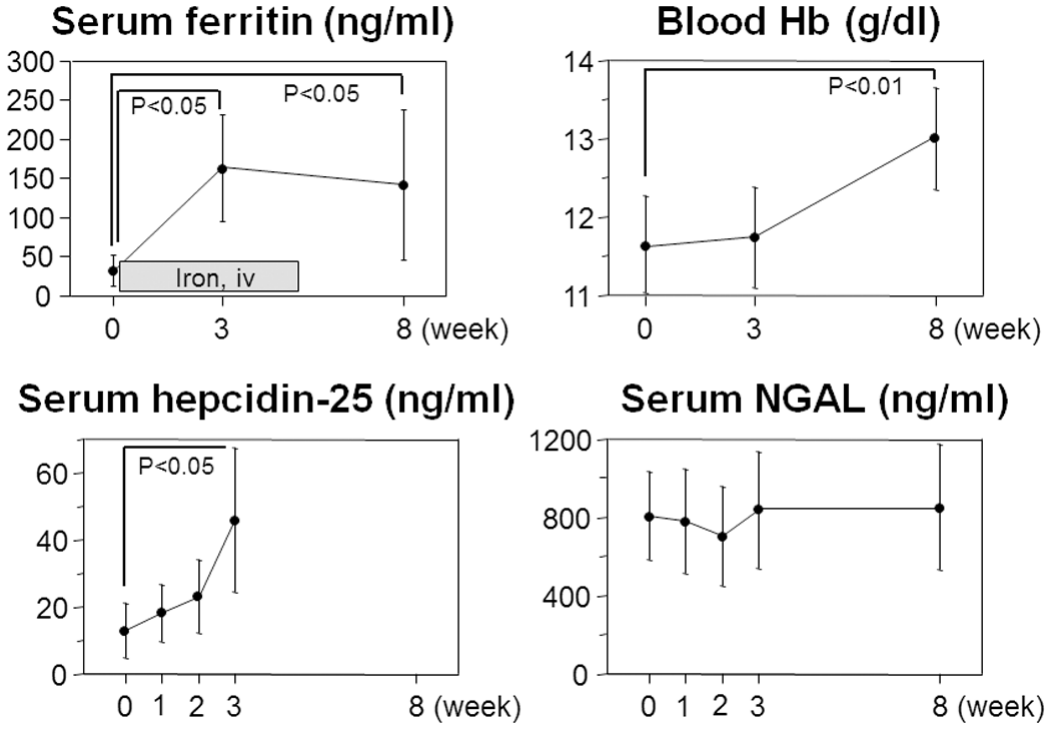
Effects of iron administration upon clinical parameters in maintenance HD patients. Mean±SD. Comparison between different time points was carried out by repeated-measures ANOVA with Dunnett post test. N = 6.

To directly test whether iron homeostasis is linked to NGAL regulation, we examined the effects of iron administration in 6 anemic HD patients who had low serum ferritin levels (33±20 ng/ml). With 10 doses of 50 mg intravenous iron injection (twice a week for 5 weeks), blood hemoglobin, serum ferritin and hepcidin-25 levels were significantly elevated at 3 or 8 weeks (Fig 3, S5 Table). Hepcidin-25 is a circulating hormone which inhibits intestinal absorption of iron and release of iron from macrophages [5, 27–29]. By contrast, serum NGAL levels were not affected during the observation period, indicating that abundance of systemic iron does not alter serum NGAL levels in HD patients.

### NGAL clearance by HD session

**Fig 4:**
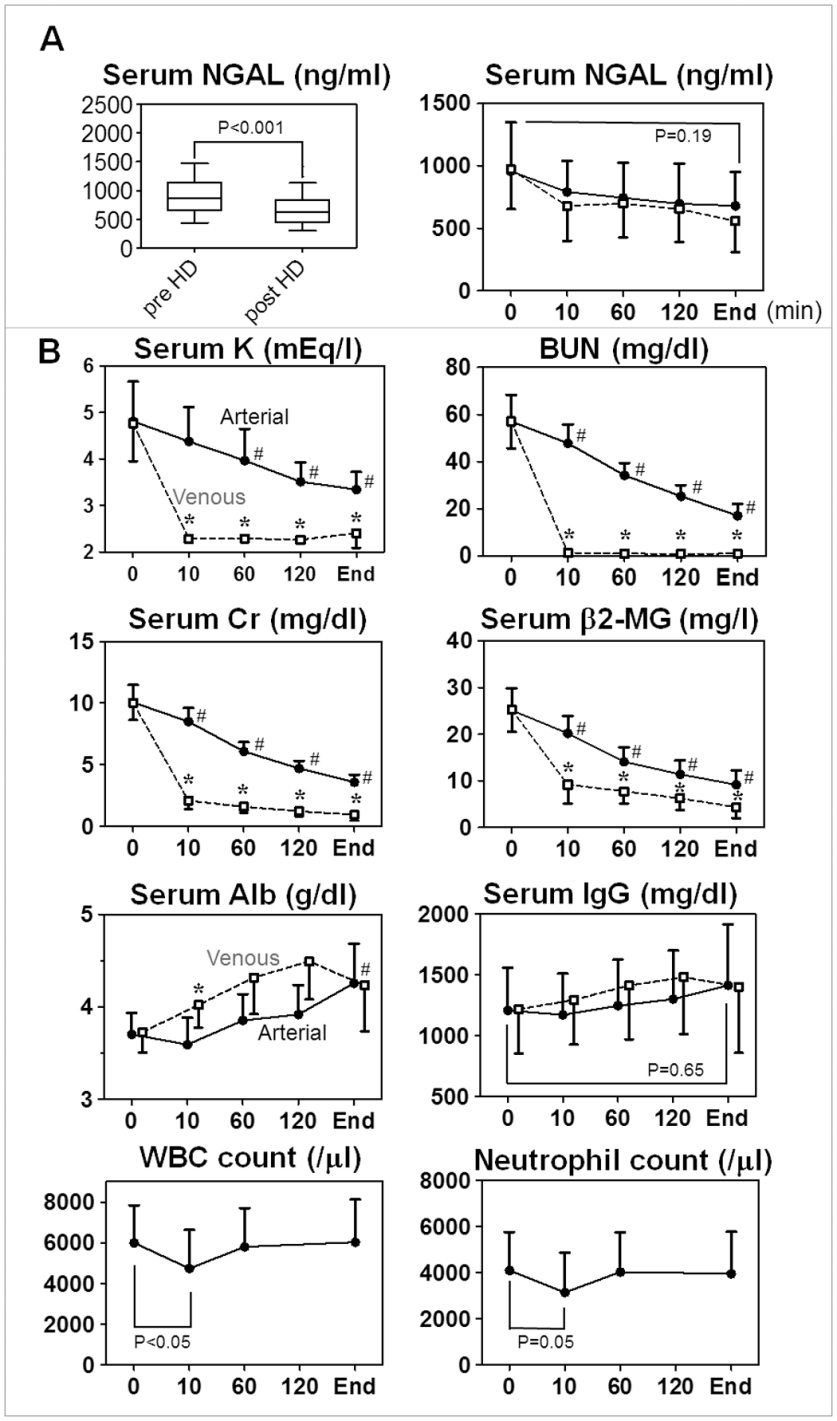
Removal of circulating substances by HD session. Blood was serially drawn from the arterial and venous ends of HD circuit (closed circle and open box, respectively) during HD session. End points varied from 3.0 to 4.5 h. Mean±SD. (**A**) Comparison of arterial blood NGAL levels between before and after HD session (left, n = 139). Time course of arterial and venous NGAL levels are also shown (right, n = 9). (**B**) Serum levels of K, BUN, creatinine (Cr), β2-microglobulin (MG), albumin (Alb) and IgG, as well as blood WBC and neutrophil counts are shown (n = 9). Arterial levels of K, BUN, Cr, β2-MG were always higher than venous levels, indicating active removal by HD. On the other hand, venous levels of albumin and IgG tended to be higher than arterial levels, reflecting hemoconcentration by HD. ^#^P<0.05, significant difference from time 0 by repeated-measures ANOVA. *P<0.05, significant difference between arterial and venous blood.

NGAL is a secretory protein whose molecular weight (MW) is 25 kilo dalton (Da) as a monomer, but it may exist in larger molecular forms, such as NGAL:matrix metalloproteinase-9 (MMP-9 or gelatinase B) heterodimer (MW 130 kDa) [1]. We examined whether circulating NGAL and other substances are removed by HD procedure (S6 Table). As shown in Fig 4A, serum NGAL level showed a rapid decrease at 10 min after initiation of HD (by 15±9%, n = 9, not significantly), which may have been caused by adsorption of NGAL to HD circuit and dialysis membrane, and it slowly decreased during the rest of time in HD. At the end of HD, NGAL levels were decreased by 27±17% (n = 139, P<0.001 comparing before and after HD; Fig 4A, Table 1). Since blood neutrophils are one of important sources of circulating NGAL [1, 30], we investigated the kinetics of blood WBC and neutrophil counts (n = 9), and observed temporal drop at 10 min after initiation of HD but it recovered to baseline by 60 min [31], indicating that neutrophil count cannot simply explain the alteration of serum NGAL levels during HD session (Fig 4B). In comparison, the arterial blood concentrations of low MW substances such as potassium ion (39 Da), BUN (60 Da), creatinine (113 Da), and β2-microglobulin (12 kDa) decreased constantly during HD session (Fig 4B). On the other hand, albumin (60 kDa) and IgG (150 kDa) levels increased slightly as water removal proceeded during HD. The kinetics of arterial blood NGAL levels were intermediate of low and high MW substances, suggesting that NGAL is constantly removed during HD but by low efficiency.

As described above, serum NGAL levels were decreased by 27% on average after HD session, but NGAL levels were rather increased in 7% of HD patients: their NGAL removal ratios were -6.2±5.4%, n = 10). NGAL removal ratio was not significantly correlated to pre-dialysis NGAL (r = 0.11, P = 0.19, n = 139) but significantly correlated to post-dialysis NGAL levels (r = -0.44, P<0.001). Univariate analysis indicated that serum HCO_3_^-^ (r = 0.37, P<0.001), base excess (r = 0.35, P<0.001), Kt/Vsp (r = 0.35, P<0.001), pCO_2_ (r = 0.30, P<0.001) and pH (r = 0.20, P<0.05) show significant correlation to NGAL removal ratios. On the other hand, unlike pre-dialysis NGAL levels, NGAL removal ratios were not significantly correlated to %CGR (r = -0.01), neutrophil count (r = 0.12) or anion gap (r = 0.00; P>0.05, respectively). These findings suggest that metabolic acidosis (low HCO_3_^-^, low base excess and low pH), secondary respiratory alkalosis (low pCO_2_) and low HD efficiency potently inhibit reduction in serum NGAL levels during HD session, independently from pre-dialysis serum NGAL levels.

### NGAL clearance by renal circulation

To study whether circulating NGAL is removed during renal passage, we collected plasma (instead of serum) from aorta and renal vein in a separate group of 30 patients who underwent coronary angiography (S7 Table). As shown in Table 4, in 15 patients not receiving HD, plasma creatinine levels in renal vein were 19±10% less compared to those in aorta (P<0.001), and plasma NGAL levels in renal vein were 13±12% less as compared to aorta (P<0.001). On the other hand, in 15 maintenance HD subjects, plasma creatinine and NGAL levels were not different between renal vein and aorta, suggesting that appreciable amount of NGAL was neither removed at nor secreted from kidneys with end-stage renal disease. As negative controls, plasma albumin (60 kDa) and choline esterase (340 kDa) [32] concentrations in aorta and renal vein were similar both in non-HD and HD subjects. These findings suggest that circulating NGAL is partially eliminated during renal passage in subjects with preserved renal function, likely by glomerular filtration [6], but not in patients with end-stage renal disease.

**Table 4:** Comparison of plasma levels of markers in aorta and renal vein among patients who underwent coronary angiography.

	Creatinine (mg/dl)		NGAL (ng/ml)		Albumin (g/dl)		Choline esterase (IU/l)	
	non-HD	HD	non-HD	HD	non-HD	HD	non-HD	HD
Aorta	0.90±0.47	7.60±1.62	92±62	765±183	4.0±0.5	3.7±0.3	274±69	216±47
Renal vein	0.73±0.39*	7.43±1.60	81±59*	768±213	4.0±0.5	3.6±0.3	275±69	212±43
(mean of relative levels)	(81±10%)*	(98±4%)	(87±12%)*	(100±10%)	(100±4%)	(99±6%)	(100±2%)	(99±5%)

Blood was collected from aorta and renal vein during coronary angiography in 15 patients not receiving HD (non-HD) and 15 receiving maintenance HD (HD).

*P<0.001 between aorta and renal vein.

### Prospective association of baseline NGAL level with serum albumin reduction and development of infection

Findings so far indicate that HD patients with low serum NGAL concentrations are characterized to have reduced muscle mass, low protein intake and mild neutropenia (Tables 1 and 3), implying morbid and infection-prone prognosis. To further elucidate clinical significance of serum NGAL concentrations in maintenance HD patients, we longitudinally investigated development of severe infection (requiring admission) and changes in serum albumin levels during a year of observation period among 95 patients in two of the three dialysis centers.

Within a year, two patients died due to congestive heart failure and lung cancer, respectively, who belonged to the middle tertile group of baseline NGAL levels (Table 5). Follow-up data could not be obtained in 6 cases (S1 Fig). Of the rest of 87 patients (S8 Table), 30 subjects (34%) underwent one to three admissions within a year. In 7 cases, treatment of infectious disease was the major purpose, at least, in one admission (among them, 4 cases belonged to the lowest NGAL tertile). Pathogens identified were *Candida albicans* (causing pneumonia), *Campylobacter fetus* (sepsis), *Enterobacter aeruginosa* (pyelonephritis), *Enterobacter cloacae* (sepsis), *Pseudomonas aeruginosa* (pneumonia) and *Streptococcus aureus* (lower extremity gangrene). In subjects with the lowest NGAL tertile, odds ratio (OR) [95% confidence interval] for development of severe infection requiring admission was 3.1 [0.6–15.0] compared to the middle and highest tertiles, but it did not reach statistical significance (S9 Table). Presence of diabetes mellitus also showed a tendency to be associated with increased occurrence of severe infection, but again not significantly (S9 Table). On the other hand, OR for admission due to non-infectious causes (23 cases, 26%) was not elevated in the lowest NGAL tertile group: 0.7 [0.2–2.0]. Non-infectious causes included diagnosis and treatment of malignancy (n = 5), cardiovascular (14), pulmonary (1), and gastrointestinal disorders (3), fixation of internal shunt (6), and ophthalmic (4) or orthopedic surgery (2), including overlaps. These findings revealed that HD patients with reduced serum NGAL concentrations show a tendency to develop severe infection.

**Table 5:** Clinical course of HD patients during a year of follow-up.

	Baseline NGAL		
	Lowest (n = 32)	Middle and Highest (n = 63)	Sum (n = 95)
Death	0	2	2
Lost follow-up	3	3	6
Follow-up available	29	58	87
Albumin loss	8	4	12
Severe infection	4	3	7
Admission without infection	6	17	23
No admission	19	38	57

Albumin Loss, reduction in serum albumin levels by 0.4 g/dl or larger after a year. Severe infection, occurrence of at least one admission within a year, in which infection was the major cause. Admission without infection, one to three times of admissions within a year, among which infection was never the major cause.

**Fig 5:**
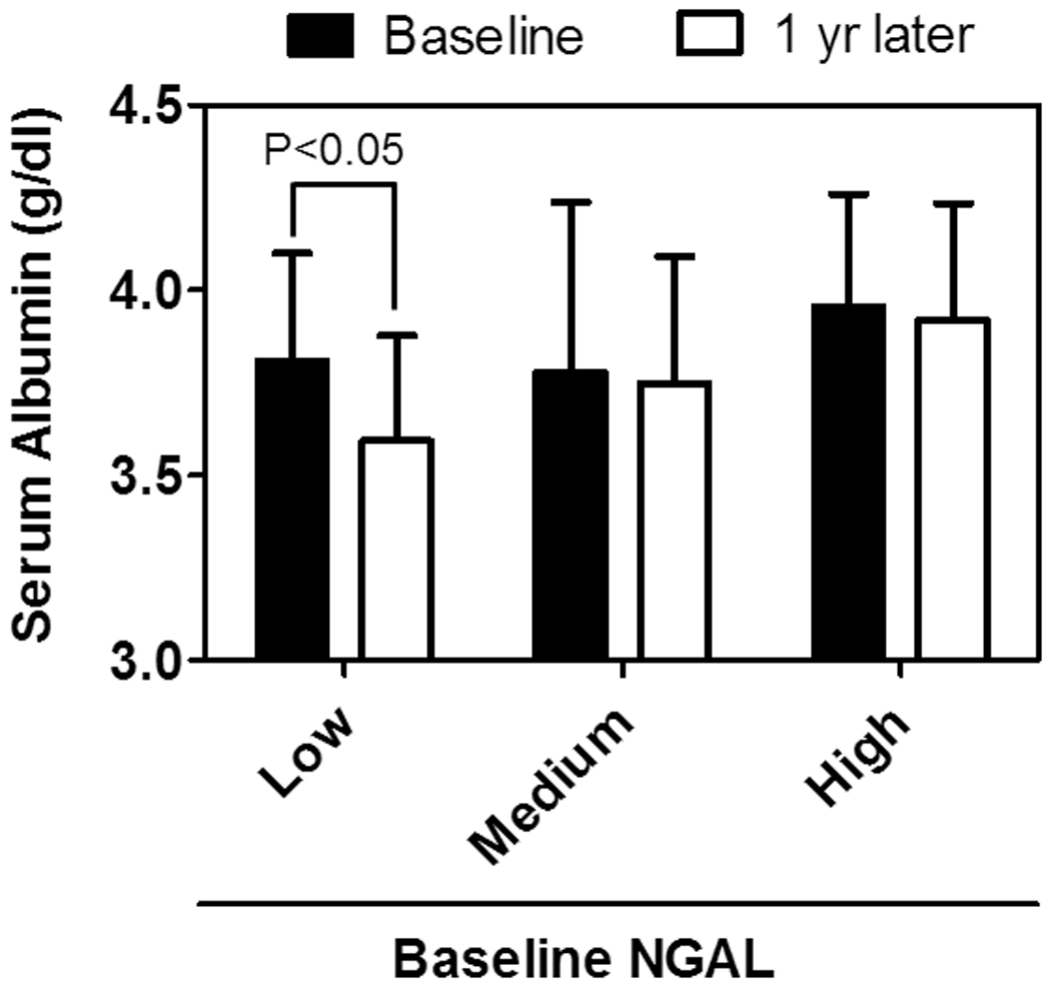
Change in serum albumin levels in a year among 3 groups assigned by baseline serum NGAL tertiles. Comparison was carried out by paired t-test. N = 87.

Next, we studied correlation of baseline serum NGAL levels with changes in serum albumin levels after a year. As shown in Fig 5, serum albumin levels were stable in the highest and middle NGAL tertiles. In the lowest NGAL tertile, serum albumin levels showed a significant decrease by 0.22±0.17 g/dl (P<0.05). Consistently, when we defined marked serum albumin decline by ≥0.4 g/dl per year (which occurred in 12 cases), its OR was 5.5 [1.5–20.3, P < 0.05] in the lowest NGAL tertile group compared to the middle and highest tertile groups (Table 6). Event numbers were too little to carry out multivariate analysis for severe infection or marked albumin loss.

**Table 6:** Prediction of marked serum albumin reduction in a year.

Variable	Odds ratio	95% CI	P
Low NGAL	5.5	1.5–20.3	0.02
DM	2.2	0.6–8.3	0.26
Male	2.2	0.6–7.8	0.23

Marked serum albumin reduction was defined as ≥0.4g/dl. Low NGAL, lowest serum NGAL tertile at baseline. DM, presence of diabetes mellitus. CI, confidence interval. N = 87.

We further evaluated relative importance of baseline serum NGAL levels among various indices of nutritional condition (Table 7). By univariate analysis, follow-up albumin levels were positively correlated to numerous nutritional markers such as baseline NGAL, albumin, geriatric nutritional risk index (GNRI) [33, 34], creatinine, %CGR, anion gap, choline esterase, triglyceride levels and neutrophil counts, and negatively to age. If not completely, similar findings were obtained for baseline albumin levels. Correlation of NGAL with follow-up or baseline albumin levels was not necessarily stronger compared to other nutritional markers. On the other hand, albumin increase was positively correlated to baseline NGAL and neutrophil but negatively correlated to baseline albumin levels. Importantly, correlation of NGAL with albumin increase (r = 0.36, P<0.01) was the strongest among markers studied in this study, suggesting that serum NGAL levels may be especially useful for the prediction of alteration in nutritional conditions among HD patients. These findings elucidated that HD patients with reduced serum NGAL levels may have increased risk to develop severe infection and large reduction in serum albumin concentrations.

**Table 7:** Correlation of baseline nutritional markers with baseline and follow-up albumin levels and with albumin increase in a year by univariate regression analyses.

Baseline variable	Baseline albumin		Follow-up albumin		Albumin increase	
	r	P	r	P	r	P
NGAL	0.19	0.09	0.41	< 0.001	0.36	< 0.01
Albumin	NA	NA	0.77	< 0.001	-0.25	< 0.05
GNRI	0.71	< 0.001	0.54	< 0.001	-0.17	0.13
Choline esterase	0.42	< 0.001	0.43	< 0.001	0.05	0.68
Creatinine	0.51	< 0.001	0.53	< 0.001	0.08	0.49
%CGR	0.42	< 0.001	0.46	< 0.001	0.06	0.57
BUN	0.10	0.35	0.14	0.19	0.04	0.69
nPCR	0.12	0.30	0.16	0.14	0.09	0.41
Anion gap	0.27	< 0.05	0.32	< 0.01	0.14	0.21
Total cholesterol	0.12	0.25	0.18	0.10	0.02	0.83
Triglyceride	0.37	< 0.01	0.40	< 0.01	0.10	0.49
Phosphorus	0.22	< 0.05	0.19	0.08	-0.03	0.81
Neutrophil	0.11	0.35	0.24	< 0.05	0.26	< 0.05
CRP	-0.16	0.16	-0.12	0.29	0.10	0.39
post-BW	0.08	0.47	0.14	0.19	0.07	0.56
post-BMI	0.04	0.73	0.04	0.75	-0.03	0.82
Age	-0.43	< 0.001	-0.46	< 0.001	-0.06	0.56
HD period	0.45	< 0.001	0.25	< 0.05	0.13	0.23
Gender	0.06	0.58	0.05	0.67	-0.04	0.74

GNRI, geriatric nutritional risk index; post-BW, post-dialysis body weight; post-BMI, post-dialysis body mass index (calculated as the weight in kilograms divided by the square of the height in meters). N = 87.

### Subanalysis after exclusion of patients with inflammatory disorders

Since inflammation, infection and malignancy have been recently reported to be associated with elevated circulating NGAL levels [9, 14, 15, 35], we performed a subanalysis by excluding such patients (S2 Table, S1 Fig). We excluded 31 subjects who had either infection (under treatment for mycobacterium tuberculosis, n = 3), malignancy (lung, colon, prostate, tonsill cancer, multiple myeloma or malignant lymphoma; n = 6) or elevated serum CRP levels (>0.5 mg/dl, n = 26), with some overlaps. Mean serum NGAL level of excluded cases (990±454 ng/ml, n = 31) were slightly (11%, P = 0.17) higher compared to the rest of patients included in subanalysis (894±305 ng/ml, n = 108). Multivariate linear regression analysis showed that baseline serum NGAL levels were determined by %CGR (β = 0.50, P<0.001) and neutrophil count (β = 0.36, P<0.001). Furthermore, the lowest NGAL tertile exhibited OR of 7.1 [1.6–29.8, P<0.01, n = 73] for marked reduction in serum albumin levels in a year, and OR of 2.9 [0.6–13.9, P = 0.22, n = 73] for development of severe infection requiring admission within a year, as compared to the middle and highest NGAL tertiles. Serum albumin increase after a year in these cases was significantly associated with baseline NGAL levels (r = 0.37, P<0.01, n = 73) and neutrophil counts (r = 0.27, P<0.05). These findings show that excluding patients in inflammatory status did not basically change determinants of NGAL levels and clinical outcomes associated with low NGAL concentrations in our study.

## Discussion

In the present study, using univariate and multivariate analyses, we have shown that serum NGAL levels in out-patient, Japanese, maintenance HD subjects were independently determined by muscle mass (represented by %CGR), nutritional status (by anion gap) and peripheral blood neutrophil counts (which presumably reflect bone marrow myeloid activity and inflammatory status). Furthermore, after one year of observation, patients in the lowest tertile of baseline serum NGAL levels were at a significant risk to experience marked decline in serum albumin levels and showed an increased tendency to develop severe infection, as compared to the middle and highest tertiles. These findings reveal for the first time, to our knowledge, that a kidney injury biomarker NGAL reflects the current nutritional status, and may be useful to predict its progressive worsening in HD patients.

In obese humans [35] and mice [36], serum NGAL levels are reportedly elevated compared to lean ones. In the present study, we propose that nutrition (by anion gap) and physical constitution (by %CGR) potently and independently affect serum NGAL levels in maintenance HD patients. In obese mice, NGAL gene expression is enhanced in the liver and adipose tissue as compared to lean mice [37]. Of note, neither pre- nor post-dialysis body mass index (BMI) was associated with pre-dialysis serum NGAL levels in HD patients of our study (S3 Table), likely because only few obese subjects were included in this study (mean BMI was 22.2 kg/m^2^), and also because congestive heart failure, hypotension and malnutrition might have caused inappropriate water retention and superficial elevation of BMI in some patients. Very recently, in the absence of kidney injury, we reported that approximately 70% of circulating NGAL derives from neutrophils in humans and mice [30]. Nutritional status is known to positively affect blood neutrophil counts [38]. As inducers of NGAL, inflammatory cytokines such as interleukin (IL)-1β and IL-6 have an activity to stimulate NGAL expression [15, 39]. Indeed, serum IL-1β levels are elevated in long-term HD patients [40], and skeletal muscle synthesizes and secretes IL-6 during HD session [41], which may play a role in massively elevated serum NGAL levels in HD patients. Through these possibilities, we speculate that neutrophils, adipocytes, liver and muscle are potential sources of circulating NGAL in HD patients. Furthermore, we elucidated that circulating NGAL is partially removed from the blood by renal passage in subjects with preserved renal function and by HD in end-stage renal disease patients.

Previous studies showed correlation between serum NGAL and CRP levels by multivariate analysis among HD patients, but significant correlation was lost in our multivariate analysis. The reasons may include (1) blood neutrophil (or WBC) counts were evaluated only in our study and contribution of CRP was incorporated into neutrophil counts, and (2) our patients had much lower CRP levels (median 0.1 mg/dl) compared to reports by Bolignano et al. (median 0.8 md/dl) [42] and Malyszko et al. (mean 0.7 mg/dl) [43].

Metabolic acidosis (low pH) compensated by respiratory alkalosis (low pCO_2_) was a typical pattern of acid-base balance in HD patients studied (S1 Table). Metabolic acidosis associated with increased anion gap may be caused by underdialysis and anorexia. On the other hand, however, high dietary protein intake (and, therefore, high nPCR) may result in greater net acid gain and acidosis (thus, high anion gap), which is not necessarily harmful in well-dialyzed HD patients [26, 44]. In this work, serum NGAL levels were positively associated with anion gap, nPCR, and Kt/Vsp [42] (Table 2), favoring the latter effect. As can be predicted from the formula for anion gap calculation (see Materials and Methods section), Cl^-^ concentration was negatively associated with anion gap (r = -0.36, P<0.001) and, thus, also with serum NGAL levels (r = -0.32, P<0.001).

Of note, serum NGAL levels were increased after HD session in 7% of patients, and metabolic acidosis was associated with elevation in serum NGAL levels. NGAL was originally identified as exocytosed material from phorbol myristate acetate-stimulated neutrophils [1]. Importantly, mild acidic environment at levels typically observed in HD patients (pre-dialysis plasma HCO_3_^-^ levels < 21 mEq/l) enhances degranulation (or oxidative burst reaction) of activated neutrophils [45], and HD procedure potentially induces neutrophil degranulation [46]. These findings raise a possibility that NGAL may be secreted from activated neutrophils during HD session.

Since NGAL is an iron carrier protein [2–4, 10, 47], it is important to test whether iron status directly affects circulating NGAL concentrations. Here we show that serum NGAL levels were not significantly altered by repeated iron administration, at the amount sufficient to cause 4.3-fold elevation in serum ferritin levels. Bolignano et al. recently reported that NGAL levels are elevated after iron injection in maintenance HD patients but only by 9% (which was accompanied with 1.8-fold ferritin elevation) [42]. The iron formula used was sodium ferric gluconate which may cause leukocyte activation by generation of reactive oxygen species [42, 48]. Indeed, there are iron formulas which reportedly do not icrease circulating NGAL levels after injection [49, 50]. Taking these findings into account, we would like to conclude that iron is not a major determinant of serum NGAL levels, at least, in maintenance HD patients. Bolignano et al. also reported marginal correlation between serum NGAL and TSAT levels (r = 0.29, P = 0.04) but it may be an indirect effect as the authors suggested [42].

In the present study, we show that maintenance HD patients with reduced serum NGAL levels were prospectively associated with decrease in serum albumin levels, and had an increased likelihood to require admission for the treatment of infectious diseases. These findings can be caused by malnutrition and neutropenia, and by reduced NGAL’s bacteriostatic activity. Growth inhibition of several but not all pathogens by iron depletion is an established activity of NGAL [10–12, 51]. Serum NGAL levels in our maintenance HD patients were approximately 10–20 fold higher compared to levels reported in healthy subjects [21, 52], but iron-chelating activity of NGAL might be partly inhibited in HD patients, since these patients are replete with exogenous iron [10] to avoid anemia (Table 1).

We further studied why baseline NGAL levels can predict future changes in albumin levels (S2 Fig). Among 87 patients who were enrolled in longitudinal analysis, correlation between baseline NGAL and increase in albumin after a year (r = 0.36, P<0.01) was much stronger than that between baseline NGAL and albumin (r = 0.19, P = 0.09). By reviewing medical records of typical cases, we realized that cases with hypoalbuminemia and high NGAL levels may experience elevation in serum albumin levels after recovery from infection (or inflammatory disorders). On the other hand, in cases with preserved albumin levels and low NGAL levels, serum albumin levels may decrease once severe infection develops and reduced albumin levels may persist even after recovery from infection. These characteristics of serum NGAL levels may make NGAL an interesting biomarker which has a robust power to predict alteration in serum albumin levels in a year.

Circulating NGAL level is an early biomarker for AKI and high NGAL concentrations predict progression of renal damage (or serum creatinine elevation) [16] and mortality in AKI [18]. On the other hand, we show here that high NGAL levels in maintenance HD patients were closely associated with good nutritional status at present and preserved serum albumin levels after a year, and therefore potentially with reduced morbidity and mortality [53]. These findings may, at first glance, appear confusing. However, similar paradoxical observations have been found for circulating creatinine level, the gold standard for renal function evaluation, and for circulating homocysteine level [54–56]. These phenomena are called reverse epidemiology or risk factor paradox.

There are several limitations in the current work. First, this is a short (one year of observation) and small scale study involving only three dialysis centers. To study clinical outcomes associated to blood NGAL levels, we examined marked changes in serum albumin levels and occurrence of severe infection, but we had to refrain from multifactor adjustments because of little event numbers. Second, in a prospective study, we examined serum albumin levels at a single point of one year after enrollment. Furthermore, hospitalized patients at enrollment were excluded in this study, which might have affected association of baseline clinical parameters with outcomes in a year. Third, mortality rate was low and a number of patients (34%) were admitted to hospitals at least once. These facts may reflect a health insurance system in Japan, in which end-stage renal disease patients are given an intense health care. Therefore, carefulness is required to extrapolate the findings obtained here into patients in other countries.

In conclusion, here we show evidence indicating that nutritional status, muscle mass and WBC counts are important determinants of serum NGAL in maintenance HD patients. It is likely that similar association also occurs in subjects with normal or nearly-normal renal function, just as serum creatinine is affected by muscle mass in patients with various renal functions [57]. Since blood and urinary NGAL levels are now vigorously measured worldwide to evaluate a broad spectrum of kidney diseases, especially in the setting of AKI, it is important to elucidate the regulatory mechanism of NGAL concentration for precise interpretation of the results from NGAL measurements. Large-scale, long-term clinical studies may be warranted to investigate whether low serum NGAL levels in maintenance HD patients are associated with higher incidence of infectious disorders, morbidity and mortality.

## Materials and Methods

### Patients

Out-patient, maintenance, Japanese HD subjects were enrolled between December 2008 through March 2009 at two dialysis centers, Saiseikai Nakatsu Hospital (n = 79) and Misugikai Satou Hospital (n = 16), in Osaka, Japan (S1 Fig). Patients were consecutively enrolled and prospectively followed for a year. New patients were added in February and March 2015 at Misugikai Otokoyama Hospital (n = 44) in Osaka, but follow up data were not available for those patients. Of overall 155 patients recruited, cases who did not agree to participate (n = 8), who were admitted during the recruitment period (n = 5), and who were under hemodiafiltration (n = 3) were omitted. Thirty-one patients under inflammatory satus were excluded in a subanalysis (as shown in the last paragraph of Results section). All patients were on HD with standard bicarbonate buffer and type III or IV high-flux membranes for 3.0–4.5 h, three times a week. Healthy volunteers were also enrolled (6 males and 2 females; age 35±5). Presence of DM was defined by taking any oral anti-DM mediaction or insulin injection. A separate group of 30 patients were enrolled at Nara Medical University Hospital, who underwent elective coronary angiography for evaluation of ischemic heart disease [58]. Only plasma was available for these patients. The protocol was approved by ethical committees in participating dialysis centers, Kyoto University Graduate School of Medicine (No. E-541), and Nara Medical University (No. 2002–009), and the study was conducted according to the Declaration of Helsinki. All participants gave written informed consent.

### Laboratory Analyses

Blood samples were collected at the beginning and end of HD session after the longest interdialytic period (on Mondays or Tuesdays). To examine serial changes in blood concentrations of NGAL and other parameters, blood was obtained also at 10, 60 and 120 min after beginning of HD in 9 patients. In 6 cases who started to be treated with intravenous iron injection (Cideferron, macromolecular complex of ferric hydroxide with dextrin and citric acid; Nippon-zoki, Osaka, Japan), blood collection was carried out at 0, 1, 2, 3 and 8 weeks after initiation of iron injection. Serum or plasma was separated immediately and kept frozen at -80°C until analysis. NGAL was measured by sandwich ELISA (BioPorto; Gentofte, Denmark) usually after 4,000-fold dilution. We studied same-patient-variability of blood NGAL levels in 10 HD patients by comparing 2 points which were 1 week apart. By paired analysis of the cases, the second measurement gave 99.6±10.6% value (mean±SD) of the first, indicating that serum NGAL levels were considerably stable, at least, for short term. Hepcidin-25 was determined at Medical Research Institute, Kanazawa Medical University by a proteomic method using surface-enhanced laser desorption ionization time of flight mass spectrometry (SELDI-TOF MS) [28]. Routine laboratory measurements were carried out in each clinical institute. Serum CRP levels were measured by an improved latex aggregation method (Nittobo Medical,Tokyo), and concentrations below 0.03 mg/dl were given a value of 0.03 (see Fig 1D). Creatinine was measured by an enzymatic method.

### Calculation of clinical indices

Calcium levels were normalized with Payne’s formula, when serum albumin was less than 4 g/dl [59]. Anion gap was determined as (Na^+^+K^+^)-(Cl^-^+HCO_3_^-^). Calculation as Na^+^-Cl^—^HCO_3_^-^ gave very similar findings (not shown). Kt/Vsp [60], nPCR [61] and %CGR [23] were determined by formulas previously described. Replacement of Kt/Vsp with equilibrated Kt/V gave almost identical results (not shown). GNRI was calculated as 14.89 x serum albumin (g/dl) + 41.7 x {(post-dialysis body weight)/(ideal body weight)}[33, 34]. When post-dialysis body weight exceeded ideal body weight, the ratio was given a value of 1.

### Statistical analyses

All variables were expressed as mean±SD, median (25–75 percentile) or number (percentage). Difference in two groups was examined either by paired t-test or repeated-measures ANOVA with Dunnett post test. Compariosn among three groups was carried out by ANOVA for continuous variables and Kruskal-Wallis test for dichotomous and non-normal variables. Univariate linear regression analysis was performed with Spearman rank test. Multivariate linear regression analysis was done with stepwise method using entrance/exit tolerances of 0.05/0.10. Odds ratio was calculated by χ^2^ test. All analyses were conducted using SPSS Version 17.0 (SPSS Inc., Chicago, IL, USA). The P values reported were two-sided and considered significant at < 0.05.

## Supporting Information

S1 Fig Overall study population. (TIF)

S2 Fig Correlation of increase in serum albumin levels after a year with baseline clinical parameters among 87 patients. Linear lines show regression lines. sAlb increase, increase in serum albumin after a year; sNGAL, baseline serum NGAL; sAlb, baseline serum albumin; sCRP, baseline serum C-reactive protein; NS, not significant. A red point indicates a 72-year-old man who had baseline concentrations of NGAL 2082 ng/ml, albumin 3.0 g/dl, CRP 4.39 mg/dl, and WBC 6120 /μl. He had mild pneumonia at enrollment. His albumin level was elevated by 0.4 g/dl in a year. A green point indicates a 72-year-old woman having baseline NGAL 414 ng/ml, albumin 4.0 g/dl, CRP 0.07 mg/dl, and WBC 2948 /μl. She developed septic shock and was admitted twice at 2 and 6 months after enrollment. She survived but hypoalbuminemia of 3.5 g/dl persisted after a year.
(TIF)

S1 Table Comprehensive baseline clinical parameters of 139 maintenance HD patients. Body mass index (BMI) was calculated as the weight in kilograms divided by the square of the height in meters. MCV, mean corpuscular volume; MCH, mean corpuscular hemoglobin; MCHC, mean corpuscular hemoglobin concentration.
(XLSX)

S2 Table Raw data of 139 HD patients at baseline. (XLSX)

S3 Table Correlations of clinical parameters with baseline pre-dialysis serum NGAL levels by univariate linear regression analysis. N = 139.
(XLSX)

S4 Table Mutual association among 5 clinical parameters which were closely correlated to baseline serum NGAL level. Numbers indicate correlation coefficients (r) for statistically significant association by univariate linear regression analysis (P<0.05). Strong correlations are highlighted in gray (P<0.001). NS, not significant. N = 139.
(XLS)

S5 Table Raw data of 6 anemic patients treated with iron. (XLSX)

S6 Table Raw data of blood concentrations of circulating substances during HD session in 9 patients. (XLSX)

S7 Table Raw data of blood concentrations of NGAL, creatinine, albumin and choline esterase in aorta and renal vein in 30 patients. AO, aorta; RV, renal vein; Cr, creatinine; ChE, choline esterase.
(XLSX)

S8 Table Raw data of clinical events which ocurred during one year follow-up in 87 HD patients. (XLSX)

S9 Table Prediction for occurrence of severe infection within a year. N = 87.
(XLSX)
