# Supplementary figures and images for: Low Serum Neutrophil Gelatinase-associated Lipocalin Level as a Marker of Malnutrition in Maintenance Hemodialysis Patients
Source: PLoS One. 2015 Jul 10;10(7):e0132539. doi: 10.1371/journal.pone.0132539 (PMC4498679; doi:10.1371/journal.pone.0132539)

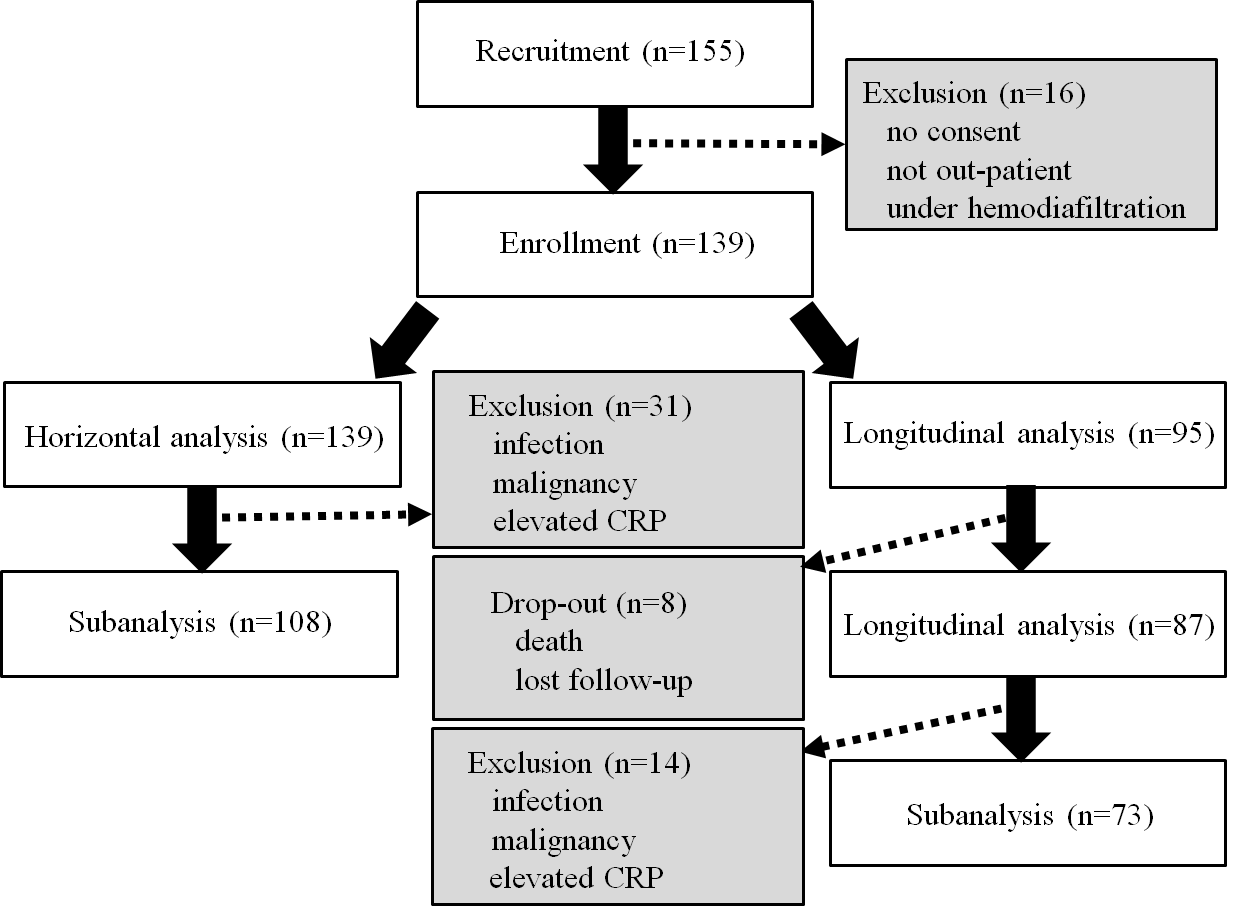

Supplement: S1 Fig — (TIF) [file pone.0132539.s001.tif]

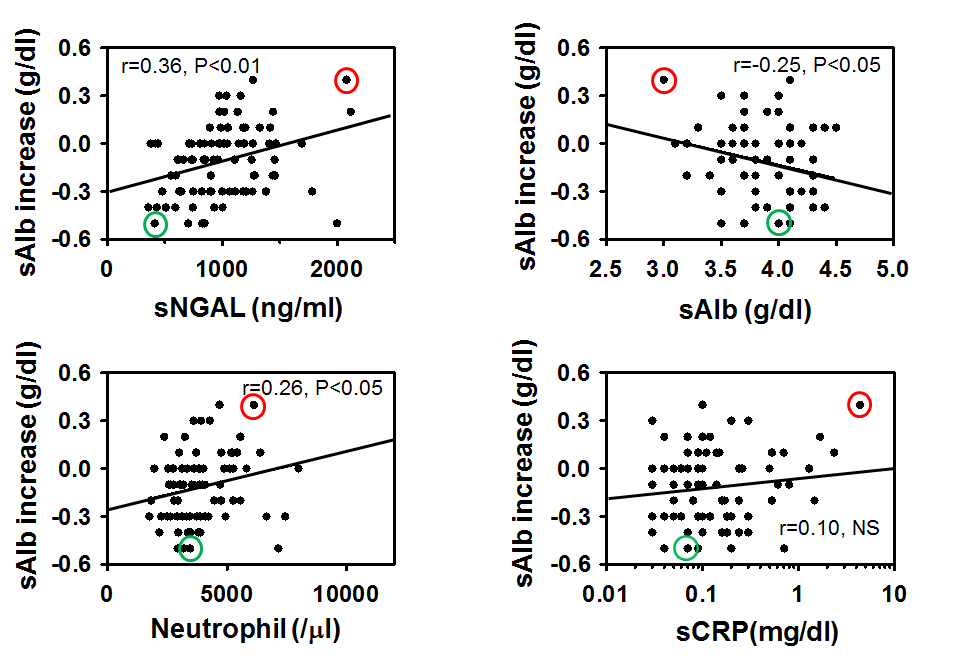

Supplement: S2 Fig — Linear lines show regression lines. sAlb increase, increase in serum albumin after a year; sNGAL, baseline serum NGAL; sAlb, baseline serum albumin; sCRP, baseline serum C-reactive protein; NS, not significant. A red point indicates a 72-year-old man who had baseline concentrations of NGAL 2082 ng/ml, albumin 3.0 g/dl, CRP 4.39 mg/dl, and WBC 6120 /μl. He had mild pneumonia at enrollment. His albumin level was elevated by 0.4 g/dl in a year. A green point indicates a 72-year-old woman having baseline NGAL 414 ng/ml, albumin 4.0 g/dl, CRP 0.07 mg/dl, and WBC 2948 /μl. She developed septic shock and was admitted twice at 2 and 6 months after enrollment. She survived but hypoalbuminemia of 3.5 g/dl persisted after a year. (TIF) [file pone.0132539.s002.tif]
